# National Kriging Exposure Estimation: Liao et al. Respond

**DOI:** 10.1289/ehp.10205R

**Published:** 2007-07

**Authors:** Duanping Liao, Donna J. Peuquet, Hung-Mo Lin, Yinkang Duan, Eric A. Whitsel, Richard L. Smith, Gerardo Heiss

**Affiliations:** Pennsylvania State University, Hershey, Pennsylvania, E-mail: dliao@psu.edu; University of North Carolina, Chapel Hill, North Carolina

Szpiro et al. suggest that our findings Liao et al. (2006) do not adequately support using national-scale, log-normal ordinary kriging to estimate daily mean concentrations of PM_10_ (particulate matter with aerodynamic diameter ≤ 10 μm) at unmonitored locations in the contiguous United States. They posit that the absence of the cross-validation SE prevents evaluating the validity of kriging estimation, as we implemented in this context, and the comparability of both regional-versus national-scale kriging and manually modified versus semiautomated, default-calculated semivariograms.

Little literature is available on the use of kriging methods to estimate daily air pollution data for large population–based multi-center epidemiologic studies. The four studies cited by Szpiro et al. (Dockery et al. 1993; Jerrett et al. 2005; Miller et al. 2007; Pope et al. 1995) all used cohort analyses for which only long-term average exposures are required, and only one of those (Jerrett et al. 2005) actually involved interpolation methods at all, although the study was restricted to a single city. In contrast, our objective was to create an interpolated daily pollutant concentration database for a multisite population-based epidemiologic study.

The cross-validation mean square error (MSE) mentioned by Szpiro et al. is also termed the “root-mean-square (RMS) prediction error,” which is the empirical SE based on the mean square of the predictions, as opposed to SE, the mathematical formula for the RMS prediction error. RMS and SE, both are available from ArcView (ESRI Inc., Redlands, CA), are often considered jointly as an alternative measure (to RMSS) of the validity of spatial analysis.

The average RMS and SE from 366 daily PM_10_ spherical model cross-validations based on year 2000 PM_10_ data were 19.48 and 16.19 μg/m^3^, respectively, from the log-normal regular kriging, and 26.43 and 25.60 μg/m^3^, respectively from a ordinary kriging. The validity of the model is supported by RMSS alone (≈1), by the similarity of RMS and SE, and by SPE (≈ 0). Additionally, the average daily SD of PM_10_ measured at the monitor locations was 27.20 μg/m^3^. Comparing SD with the kriging-RMS provides a measure of the reduction in error due to interpolation. If RMS is less than the SD, then the kriging approach has some benefit, compared with using long-run averages. From both ordinary and log-normal kriging, especially for the latter, we see a notable reduction in RMS compared with SD. Meanwhile, substantial variability remains, suggesting that kriging error should be taken into account when using the kriged values.

Szpiro et al. also implicitly criticize our use of daily kriging when the objective was to interpolate daily data. Spatial–temporal models have potentially greater power than a 1-day-at-a-time spatial analysis but are not easy to apply in practice, with large datasets and many missing values.

Regional kriging could be superior to national kriging if the spatial dependence parameters (range, sill, and nugget) vary substantially from region to region, in which case a national kriging model could result in misspecified covariances. However, regional kriging also uses fewer data points to estimate those parameters and could result in greater errors. We would welcome theoretical or empirical studies that could cast further light on this trade-off. However, as far as our article (Liao et al. 2006) is concerned, our main purpose was to note that the national kriging method appears to be competitive when assessed by overall RMS error. We compared the results of regional- and national-scale kriging on a small set (17%) of days when the largest number of monitors (≥ 400) were reporting data—a scenario heavily favoring regional spatial interpolation strategies. On the remaining days when only 120–400 monitors were reporting data, regional kriging was inherently problematic given the restricted availability of monitors within regions. Szpiro et al. suggest that the problems of interpolation near the boundary could be solved by “overlapping,” but this is only one of the issues encountered using regional-kriging: One would still need to decide how to consistently define the regions, considering the number of available data points that change substantially from day to day, to achieve a meaningful reduction in RMS error.

Based on the 12 “optimal” days in 2000, the average RMS and SE were 12.68 and 12.82 μg/m^3^, respectively, from the national scale kriging, compared with 12.22 and 12.49 μg/m^3^, respectively, from regional-scale kriging (Liao et al. 2006). These results, together with RMSS and SPE we reported, support our conclusion that national kriging performs comparably to regional kriging even when restricted to optimal days.

Szpiro et al. correctly note that it is possible to improve the RMSS values by manual adjustment. However, typically we found that when one of the validity measures (RMSS, PE, or SPE) was improved by manual adjustment, other measures became worse. It is difficult to manually adjust models to improve all cross-validation parameters simultaneously. Manually adjusting daily semivariogrms is not feasible when kriging over 10 years. Moreover, the predicted SE at unmeasured locations was uniformly lower in automatically fit models.

Szpiro et al. are correct that cross-validation may not be representative of the performance at participant address locations, although it is unclear what alternative methods they would like us to use. The ability to do semi-automatic cross-validations was a major attraction of ArcView and, despite limitations, is the best tool we know for validating spatial predictions.

The semiautomated kriging approach presents considerable advantages in estimating daily residential-level pollutant concentrations in large cohorts over long periods. Our proposed method (Liao et al. 2006) used log-normal kriging based on a spherical model to interpolate daily data on a national scale, and the weighted least squares method of parameter estimation without manual adjustment. We believe that the cross-validation statistics, presented in our article and amplified here, provide adequate support for these recommendations against reasonable alternatives that we considered.
